# Manifestations of psychological distress in students from different social groups at the University of São Paulo

**DOI:** 10.1590/1980-220X-REEUSP-2024-0231en

**Published:** 2025-02-21

**Authors:** Enola Julio Mango, Celia Maria Sivalli Campos, Cassia Baldini Soares, Carla Andrea Trapé

**Affiliations:** 1 Universidade de São Paulo, Escola de Enfermagem, Departamento de Enfermagem em Saúde Coletiva, São Paulo, SP, Brazil.

**Keywords:** Mental Health, Social Class, Students, Universities, Salud Mental, Clase Social, Estudiantes, Universidades

## Abstract

**Objective:**

To analyze the distribution of manifestations of psychological distress in different social groups of undergraduates from health courses at the University of São Paulo.

**Method:**

A cross-sectional study was carried out with undergraduates from 11 health courses at the University of São Paulo. The non-probabilistic sample consisted of 108 students. Data was collected using an online form and analyzed using the SPSS 20.04 and STATA 17 statistical packages. The students were classified into three social groups using the Social Reproduction Index.

**Results:**

188 students took part in the study, 77.4% female; 64.7% white; 69.7% lived in the city of São Paulo and only 4.8% of these lived in student housing. As for the Social Group (SG), 50.5% were classified as SGI, 26.1% as SGII and 23.4% as SGIII/GSIV. Most of the university respondents (77.7%) reported manifestations of psychological distress. The GSI students expressed a lower percentage of manifestations of psychological distress (62.1%) when compared to the other groups.

**Conclusion:**

there was a higher prevalence of psychological distress among undergraduates in the GSIII/GSIV groups. The study highlights the importance of implementing policies to deal with psychological distress among university students.

## RESUMEN

**Objetivo:**

Analizar la distribución de las manifestaciones de malestar psicológico en diferentes grupos sociales (GS) de estudiantes de pregrado en salud de la Universidad de São Paulo.

**Método:**

Estudio transversal con estudiantes de pregrado de 11 cursos de salud. Los datos fueron colectados por medio de formulario online y analizados con SPSS 20.04 y STATA 17. Los estudiantes fueron clasificados en tres GSs utilizando el Índice de Reproducción Social.

**Resultados:**

Hubo 188 participantes, 77,4% mujeres; 64,7% blancos; 69,7% vivían en la ciudad de São Paulo; 50,5% fueron clasificados en GSI, 26,1% en GSII y 23,4% en GSIII/GSIV. La mayoría (77,7%) relató manifestaciones de malestar psicológico, con el menor porcentaje en GSI (62,1%).

**Conclusión:**

La mayor prevalencia de malestar psicológico se registró entre los estudiantes universitarios de los grupos GSIII/GSIV. El estudio destaca la importancia de implementar políticas de abordaje del malestar psicológico entre los universitarios.

## INTRODUCTION

The subject of this study is the distribution of manifestations of psychological distress in different social groups among undergraduates at the University of São Paulo (USP). Mental health problems have taken on a worrying dimension, which needs to be tackled within the public health sphere^(1)^. There has been an increase in cases of psychological distress, especially in the post-pandemic context^(2)^, with a particular emphasis on manifestations such as anxiety and depression, associated with the dynamics of the contemporary mode of production^(1)^. This reality suggests the need to pay attention to the roots of this suffering.

Suicide, the most disastrous consequence of these conditions, happened in Brazil in more than 147.000 cases between 2011 and 2022, with the highest percentage among young people^(3)^. In this context, the mental health of university students is an urgent social issue and is gaining ground in academic discussions. The results of a survey carried out at 19 universities in countries on several continents found that 31% of new students had at least one disorder in 12 months, with depression and anxiety being the most prevalent^(4)^.

A Brazilian survey carried out by the National Forum of Pro-Rectors of Community and Student Affairs (FONAPRACE)^(5)^ with 424,128 undergraduates from federal universities identified that 63.6% of respondents suffered from anxiety; 45.6% from discouragement/demotivation; 32.7% had insomnia/alterations in sleep; 28.2% felt helpless/despairing; 23.5% reported feeling lonely; 22.9% persistent sadness; 22.1% inattention/disorientation/mental confusion; 13.5% feelings of fear/panic; 12.3% eating problems; 10.8% death ideation and 8.5% suicidal thoughts. The relationship between the heterogeneous distribution of mental health problems and belonging to different social classes has been well analyzed in epidemiological research concerned with the theoretical dimension of social classes, analyzing fragmented empirical social data as expressions of class structure^(6)^.

Consistent with the conditions of contemporary social reproduction, this study adopted an understanding of psychological suffering as a consequence of the characteristic socialization processes that produce obstacles to the fulfillment of life. However, they are not always pathological, such as the suffering caused by bereavement, as long as the individual finds the resources to maintain the conditions to live their daily life^(1)^. Therefore, the increasing percentages of psychological suffering, especially anxiety and depression, are attributed to the characteristics of how life is socially produced^(1)^. Human nature is not natural, it is permeated with socially produced values and one of the founding contemporary values is competition, which requires a lack of responsibility towards others and the extolling of the morality of hedonism. Contemporary ideology praises illusory self-sufficiency, causing individuals to oscillate between helplessness and omnipotence^(7)^. This study is based on the understanding that psychological suffering is the result of the experiences of life in society, which are heterogeneous, unequal and consistent with the characteristics of social insertion. It considers experiences among people and between people and institutions^(8,9)^, including universities, which in Brazil have become home to a greater social heterogeneity of entrants since the implementation of affirmative policies. USP was the last of the public universities to implement these affirmative policies, and its social heterogeneity of undergraduates can be confirmed by the percentage of entrants from public schools; in 2024 there were 5.954 students (55.4% of all entrants), 2.965 (27.6%) of whom self-declared black, brown or indigenous^(10)^.

Against this background, this study assumed that manifestations of psychological distress are distributed heterogeneously among undergraduates from different social groups, and aimed to analyze the distribution of these manifestations in the different social groups of undergraduates from health courses at USP.

## METHOD

The general outline of the text followed the recommendations of STROBE - Strengthening the Reporting of Observational Studies in Epidemiology^(11)^.

### Study Design

This is a cross-sectional study, grounded on critical epidemiology, which studies the heterogeneous distribution of diseases and illnesses in line with the social reproduction conditions of social classes/groups^(12)^. This study presents the manifestations of psychological distress among undergraduates from health courses at USP, which is part of the results of a larger study which included undergraduates from other areas offered by USP.

### Study Site

Data collection followed the steps of the *Checklist for Reporting Results of Internet E-Surveys* (CHERRIES), especially with regard to data protection^(13)^. Location: USP, with around 60.000 undergraduates and 12 health courses in the city of São Paulo (physical education and health, nursing, pharmacy, physiotherapy, speech therapy, gerontology, medicine, nutrition, obstetrics, dentistry, public health, occupational therapy). Data was collected in a convenience sample between January and August 2022, following an invitation sent by email by the undergraduate secretariats to all students on the courses, and also by students on the courses in WhatsApp groups. The link to the form was made available on both communication channels. Data entry was tracked by monitoring responses on the *Google Forms* survey management application.

### Participants

The only criterion to be included in the study was to be an undergraduate student enrolled in one of the 12 health courses at USP, who agreed to fill in the data collection instrument in the *Google* Forms application.

### Data source

Data collection used a self-administered form, with three structured questionnaires, one of which was the social classification of families, with questions that make up the Social Reproduction Index (SRI)^(14)^, the result of methodological research, with statistical validation, already used for social classification of families in other studies^(15,16)^.

The SRI is based on the understanding that social classes reproduce themselves in order to maintain social relations; therefore, they have particular working (production) and living (reproduction) conditions. The IRS captures conditions considered relevant today and the classification goes from the SGI to the SGIV social group, ranging from the highest (SGI) to the lowest (SGIV) stability in families’ working and living conditions (social reproduction conditions). As an example of the working and living conditions variables that make up the indicator, in the SGI the majority of heads of household are involved in activities related to planning, management and direction, occupations that require preparatory training for work and almost all of them own their own homes; in the SGII the heads of household are involved in occupations that do not require technical training for work and a considerable proportion live in rented homes; in SGIII, the majority of heads of household are not in the labor market, most are retired, unemployed or on INSS leave, and live in their own homes; in SGIV, families live in more unstable working and living conditions, with heads of household working in general services, which don’t require a formal job preparation course, sometimes requiring training to perform the activities. They live in their own homes, in precarious conditions, almost all of which are not connected to the official sewage system^(14)^.

After pre-testing, in order to facilitate self-completion, captions with the Brazilian Classification of Occupations were added to the SRI form, to make it easier to identify the classification of occupations of the heads of households.

The second questionnaire was the Self Reporting Questionnaire-20 (SRQ-20), with 20 questions on expressions/manifestations of psychological distress. This is an internationally standardized instrument recommended by the WHO for its sensitivity and specificity^(16)^. The third questionnaire, with 12 questions on expressions/manifestations of psychological distress, has been systematically used in surveys, obtaining results from hundreds of thousands of undergraduates at federal universities across the country, by FONAPRACE^(5)^.

### Variables

In order to characterize the respondents, the following variables were chosen: age, gender, place of residence, color/ethnicity, university admission process (entrance test, Enem/SISU grades, PEC-G agreement, transfer), course, year of commencement, whether or not they received a scholarship from student permanence support programs and whether they are the first generation in their family to attend higher education.

To characterize the SRI: job qualifications of the head of the family, completion of a preparatory course for work, (in)formality at work or unemployment; home ownership, legal access to water, sewage and electricity, number of rooms to sleep in, payment of municipal taxes and going to worship as a form of leisure.

To identify manifestations of psychological distress, the same variables used by FONAPRACE (2019) were adopted: anxiety; persistent sadness; excessive shyness; fear/panic; insomnia or significant changes in sleep; feeling of helplessness/despair/desperation; feeling of inattention/disorientation/mental confusion; eating problems; discouragement/lack of desire to do things; feeling of loneliness; idea of death, and suicidal thoughts^(5)^ and the *Self Reporting Questionnaire-20* (SRQ-20) variables were adopted, regarding the presence of physical and psychological symptoms, containing a dichotomous scale (yes/no) for each of its questions^(17)^.

### Sample

The sample size was determined based on the relationship between manifestations of psychological distress and social group, considering the population of the research that gave rise to this study. The effect size was based on information from the pilot sample of 33 undergraduates. In this sample, a W value of 0.65 was observed; aiming for a more conservative sample, a value of W = 0.40 was adopted. Thus, a sample of 108 undergraduates was calculated for each of the 4 areas (Health, Biological Sciences, Human Sciences and Exact Sciences), totaling an effective sample of 432 students, to detect an effect size of 0.4, in a Chi-square test with 3 degrees of freedom at a significance level of 5% and power of 95%. The sample is not probabilistic. Based on this calculation, a sample of 108 undergraduate health students was defined for this study.

### Quantitative Variables

Logistic regression models were adjusted, with the dependent variable being manifestation of psychological distress and the explanatory variables being gender, age, color, city, residence at USP, year of starting the course and social group. In the logistic regression model, the exponentiated coefficients are interpreted as odds ratios. In this study, the odds ratio is the quotient between the probability of a student showing signs of psychological distress and the probability of not showing such a condition.

### Statistical Methods

The analysis was carried out using the SPSS 20.0 4^1^ and STATA 17^2^ statistical packages. The overall internal consistency between the SRQ20 items was analyzed using the Cronbach›s Alpha coefficient; the closer to 1 the greater the consistency between the items of the scale or domain. A significance level of 5% was used for all statistical tests.

The expected result is a heterogeneous distribution of psychological distress among students from different social groups, with a greater likelihood of manifestations among students from social groups with more unstable working and living conditions of their families.

### Ethical Aspects

The study was cleared by the Research Ethics Committee (opinion no. 246/2021/CPq/EEUSP). After formally agreeing to the Informed Consent Form, participation took place online, by filling in an electronic form. At the end of the form there was a link to USP’s mental health resource map, should the respondent feel the need for help (https://mapadesaudemental.prip.usp.br/).

## RESULTS AND ANALYSIS

There were 188 respondents from 11 courses, with no participants from the physical education and health course. Of these, 144 (77.4%) were female and 121 (64.7%) were white. The average age of the respondents was 21.5 years (SD = 4.9 years), with a minimum age of 17 and a maximum of 65, of which 94 (50%) were aged between 19 and 22.In terms of place of residence, 131 (69.7%) lived in the city of São Paulo and only 9 (4.8%) of the respondents lived in the USP residential complex (CRUSP).

In terms of social classification, 95 (50.5%) of the families were classified in social group I (SGI), where the heads of household have more stable working and living conditions, compared to the other three social groups (SG). There were 49 (26.1%) respondents in social group II (SGII), 26 (13.8%) in social group III (SGIII) and 18 (9.6%) in social group IV (SGIV). Due to the lower percentage of families classified as SGIII and SGIV, they were grouped together, corresponding to 23.4% (44) of the respondents. The same strategy was used by a study that analyzed child development in different social groups^(18)^.

The analysis identified 133 (70.7%) respondents with manifestations of psychological distress and found an association between SG and manifestations of psychological distress (p = 0.031). Among SGII respondents, 149 (79.6%) had psychological distress, the same number, 149 (79.5%), among respondents from SGIII/SGIV families and 116 (62.1%) among SGI respondents, as can be seen in Table 1.

**Table 1:** Distribution of psychological distress by characteristics and social groups of USP health course students – São Paulo, SP, Brazil, 2022.

	Psychological distress (SRQ-20 ≥ 7)		p
	No	Yes	
**Gender, N (%)**			0.496^a^
Feminine	39/144 (27.1)	105/144 (72.9)	
Masculine	14/39 (35.9)	25/39 (64.1)	
Non-binary	1/3 (33.3)	2/3 (66.7)	
**Age (years), Mean ± SD**	21.9 ± 7.6	21.4 ± 3.2	0.610^c^
**Color, ethnicity, N (%)**			0.436
White	40/121 (33.1)	81/121 (66.9)	
Brown	7/35 (20.0)	28/35 (80.0)	
Black	4/18 (22.2)	14/18 (77.8)	
Yellow/indigenous	4/13 (30.8)	9/13 (69.2)	
**City, N (%)**			0.107
Sao Paulo Municipality	40/131 (30.5)	91/131 (69.5)	
Other municipalities in the Sao Paulo state	9/45 (20.0)	36/45 (80.0)	
Municipalities in other Brazilian states	6/12 (50.0)	6/12 (50.0)	
**Living in the university, N (%)**			0.288^a^
No	54/179 (30.2)	125/179 (69.8)	
Yes	1/9 (11.1)	8/9 (88.9)	
**Year the course started**			0.099
2018	7/27 (25.9)	20/27 (74.1)	
2019	6/28 (21.4)	22/28 (78.6)	
2020	8/31 (25.8)	23/31 (74.2)	
2021	7/37 (18.9)	30/37 (81.1)	
2022	27/65 (41.5)	38/65 (58.5)	
**Social Group, N (%)**			0.031
SGI	36/95 (37.9)	59/95 (62.1)	
SGII	10/49 (20.4)	39/49 (79.6)	
SGIII/SGIV	9/44 (20.5)	35/44 (79.5)	

p-descriptive level of the Chi-square test

Fisher’s exact test(^a^)

Student’s t test(^c^).

This association was not statistically significant for the categories color/ethnicity, age and gender, city of origin, living in university housing and psychological distress, as can be seen in Table 1. The variables gender and color/ethnicity were not answered by all the respondents.

After adjusting for logistic regression, membership of the SG remained significant in the final model (p = 0.034). As a result, the chances of psychological distress (SRQ20 ≥ 7) among undergraduates in the SGII and SGIII/SGIV groups were similar; however, they were higher than among those in the SGI group (around 2.4 times higher), as shown in Table 2.

**Table 2:** Chance of students in health courses at USP experiencing psychological distress according to gender, age, color, city, living in student housing, year of starting the course and social group − São Paulo, SP, Brazil, 2022.

	Univariate model		Multivariate initial model		Final model	
	OR (CI95%)	p	OR (CI95%)	p	OR (CI95%)	p
**Gender (ref. Feminine)**		0.555		0.931		−
Masculine	0.66 (0.31−1.40)	0.284	0.91 (0.38−2.15)	0.822	−	−
Non-binary	0.74 (0.07−8.42)	0.810	0.66 (0.05−8.88)	0.751	−	−
**Age (years)**	0.98 (0.92−1.04)	0.494	0.95 (0.88−1.02)	0.157	−	−
**Color/ethnicity (Ref. = White)**		0.444		0.610	−	−
Brown	1.98 (0.79−4.91)	0.143	1.95 (0.71−5.34)	0.192	−	−
Black	1.73 (0.53−5.59)	0.361	1.25 (0.32−4.82)	0.749	−	−
Yellow/Indigenous	1.11 (0.32−3.83)	0.867	0.88 (0.23−3.33)	0.847	−	−
**City (ref. = Municipality of Sao Paulo)**		0.119		0.177	−	−
Other municipalities in the Sao Paulo state	1.76 (0.77−3.99)	0.177	2.05 (0.85−4.97)	0.111	−	−
Municipalities in other Brazilian states/other countries	0.44 (0.13− 1.45)	0.176	0.60 (0.14−2.58)	0.488	−	−
**Living in the University**	3.46 (0.42−28.31)	0.248	3.05 (0.31−29.81)	0.337	−	−
**Year the course started (ref. = 2019)**		0.109		0.077		−
2015 a 2018	0.78 (0.22−2.71)	0.695	0.72 (0.17−3.01)	0.653	−	−
2020	0.78 (0.23−2.63)	0.693	0.64 (0.15−2.64)	0.535	−	−
2021	1.17 (0.35−3.96)	0.802	0.89 (0.21−3.74)	0.871	−	−
2022	0.38 (0.14−1.07)	0.068	0.25 (0.07−0.92)	0.036		
**Social group (ref. = SGI)**		0.034		0.132		0.034
SGII	2.38 (1.06−5.34)	0.036	2.17 (0.87−5.43)	0.097	2.38 (1.06−5.34)	0.036
SGIII/SGIV	2.37 (1.02−5.51)	0.044	2.16 (0.81−5.73)	0.122	2.37 (1.02−5.51)	0.044

N = 184 and N = 188, respectively for the initial and final multivariate models.

Hosmer and Lemeshow goodness of fit test: initial (p = 0.860) and final (p = 1.000) multivariate models.

Statistical significance was found for the association between SG and feelings of excessive shyness (p = 0.046) and between SG and feelings of helplessness/despair (p = 0.026).

In SGII and SGIII/SGIV the highest percentages were related to feelings of excessive shyness, 37.6% (35 respondents), while in SGI excessive shyness was reported by 24.2% (23 respondents). The feeling of helplessness/despair was mentioned by 64.5% (60 respondents) of the SGII and SGIII/SGIV groups and by 48.4% (46) of the SGI.

## DISCUSSION

Respondents in this study were predominantly female, white and from SGI families. The predominance of females on health courses has also been identified in other studies^(19,20,21)^.

The white majority profile found in this study follows the national trend in universities, which have around 20% more white students than Brazilian society as a whole^(22)^. The results of a study carried out at three federal universities in Minas Gerais found that, although the majority were white in health courses, the majority were non-white in Biomedicine, Physical Education and Nursing^(22)^.

In this study’s sample, families classified as SGI and SGII together accounted for 76.6% of respondents, which confirms that although affirmative policies have been adopted in Brazil, the expansion of places in higher education still does not guarantee social equality in access^(23)^. Admission to university is still marked by profound inequalities, with young people from more stable social classes having much better chances^(24)^.

Furthermore, the difficulties do not end when entrying to the university; an association has been identified between social origin and a sense of belonging, the quality of the university experience and characteristics of university adaptation^(25)^. University is also a social space for experiencing psychological distress^(26)^ because, as a social institution, it reproduces hegemonic social values. In this way, while entering public universities is a potential source of strength for families, it is also a potential source of wear and tear and suffering for undergraduates.

The percentage of undergraduates with a feeling of excessive shyness and a sense of helplessness/despair was higher among undergraduates from the less stable groups (SGII, SGIII/SGIV), when compared to respondents from the SGI, a more stable social group.In this context, the university needs to prepare itself to receive undergraduates with heterogeneous social conditions. What we have seen, on the contrary, are undergraduates entering an institution that values a meritocratic culture, which produces a feeling of incapacity^(27)^.

Although it is undeniable that going to the university is potentially strengthening for undergraduates and their families, it also represents the potential for wear and tear, which is more intense for those from social groups with greater instability. Added to this is the finding in this study that the possibility of having support networks decreases as you move from SGI to SGIV, with SGI students having the largest support network, also identified in other studies^(14,28)^ as strengthening mental health.

Presently, daily university life reproduces hegemonic social values, such as competitiveness and individual success, which produce a feeling of helplessness^(8)^, as opposed to collective experiences and solidarity, which reinforce a sense of belonging. However, the university is in a position to propose actions to confront this logic of praising individual achievements^(18)^, in order to tackle the psychological suffering of students beyond its individual dimension, based on the social processes that give rise to it^(9)^.

University policies can be used to deal with the psychological distress of undergraduates: to establish and support the strengthening of mental health; to identify students’ mental health needs and respond to them, within the scope of the courses; to integrate and expand the offer of mental health care programs and provide students with access to them; to promote educational programs and communication strategies on psychological distress in contemporary times and ways of coping, so that students can find strengthening practices; constant monitoring and continuous evaluation of students’ mental health needs^(29)^. For university students to be fully integrated into academic life, they must have solid support structures, involving all institutional sectors. The importance of student sociability should be part of the training of university workers^(30)^.

## CONCLUSION

The results of this study confirmed the assumption that there is a heterogeneous distribution of psychological distress, as attested to by the SQR-20. The results showed that students in the SGII and SGIII/SGIV groups were around 2.4 times more likely to have symptoms of psychological distress than the SGI group. The feeling of excessive shyness and a sense of helplessness/despair were more recurrent in the SGII and SGIII/SGIV groups.

Although USP has been implementing actions in response to needs related to psychological distress, such as the ECOS project (Listening, Care and Guidance in Mental Health), among other actions by the Pro-Rectorate for Inclusion and Belonging (PRIP), in order to deepen existing initiatives, we advocate prioritizing the university’s material and immaterial resources to improve the infrastructure of housing and educational institutions; to improve the articulation between the actions implemented to deal with psychological suffering, starting with processes to identify students’ SG and the manifestations of suffering, to strengthen projects aimed at prevention and care, taking as a premise the roots of the production of suffering.

The study’s limitation lies in possible bias, since the sample was constituted by convenience; therefore, there may have been a tendency for responses from undergraduates mobilized by the theme because they were experiencing psychological distress.

This study was limited to investigating health students. In order to broaden the analysis, studies are needed among undergraduates from other areas.

## Footnotes

1 IBM Corp. Released 2011. IBM SPSS Statistics for Windows, Version 20.0. Armonk, NY: IBM Corp.

2 StataCorp. 2021. Stata Statistical Software: Release 17. College Station, TX: StataCorp LLC.
