# Supplementary material for: Manifestations of psychological distress in students from different social groups at the University of São Paulo
Source: Rev Esc Enferm USP. 2025 Feb 21;58:e20240231. doi: 10.1590/1980-220X-REEUSP-2024-0231en (PMC11849675; doi:10.1590/1980-220X-REEUSP-2024-0231en)
Supplement: Translation

# Translation: Manifestações de sofrimento psíquico em estudantes de diferentes grupos sociais, na Universidade de São Paulo

**DOI:** 10.1590/1980-220X-REEUSP-2024-0231pt

**Authors:** Enola Julio Mango, Celia Maria Sivalli Campos, Cassia Baldini Soares, Carla Andrea Trapé

**Affiliations:** 1 Universidade de São Paulo, Escola de Enfermagem, Departamento de Enfermagem em Saúde Coletiva, São Paulo, SP, Brasil.

**Keywords:** Saúde Mental, Classe Social, Estudantes, Universidades

## Abstract

**Objetivo:**

Analisar a distribuição das manifestações de sofrimento psíquico em diferentes grupos sociais (GS) de graduandos da área da saúde da Universidade de São Paulo.

**Método:**

Estudo transversal com graduandos de 11 cursos da área da saúde. A coleta foi por meio de formulário on-line e a análise utilizou o SPSS 20.04 e STATA 17. Os estudantes foram classificados em três GS, utilizando-se o Índice de Reprodução Social.

**Resultados:**

Foram 188 participantes, 77,4% do sexo feminino; 64,7% de cor branca; 69,7% residiam no município de São Paulo; 50,5% foram classificados no GSI, 26,1% no GSII e 23,4% no GSIII/GSIV. A maior parte (77,7%) referiu manifestações de sofrimento psíquico, com menor porcentagem no GSI (62,1%).

**Conclusão:**

A maior prevalência de sofrimento psíquico foi entre graduandos do GSIII/GSIV. O estudo destaca a importância da implementação de políticas para o enfrentamento ao sofrimento psíquico de universitários.

## INTRODUÇÃO

O objeto deste estudo é a distribuição das manifestações de sofrimento psíquico em diferentes grupos sociais, entre graduandos da Universidade de São Paulo (USP).

Os problemas de saúde mental vêm tomando dimensão preocupante, a ser enfrentada no âmbito da saúde pública^(1)^. Observa-se aumento de quadros decorrentes de sofrimento psíquico, especialmente no contexto pós-pandemia^(2)^, com destaque às manifestações expressas como ansiedade e depressão, associadas às dinâmicas do modo de produção contemporâneo^(1)^. Essa realidade sugere a necessidade de atenção ao que está nas raízes desse sofrimento.

No Brasil o suicídio, a consequência mais desastrosa desses quadros, teve registro de mais de 147 mil casos entre 2011 e 2022, com o maior percentual entre a população juvenil^(3)^.

Nesse contexto, a saúde mental de universitários é pauta social urgente e vem ganhando espaço nas discussões acadêmicas. Resultados de pesquisa realizada em 19 universidades, em países de vários continentes, identificaram que 31% dos ingressantes apresentaram ao menos um transtorno em 12 meses, sendo depressão e ansiedade os mais prevalentes^(4)^.

No Brasil, pesquisa realizada pelo Fórum Nacional de Pró-Reitores de Assuntos Comunitários e Estudantis (FONAPRACE)^(5)^ com 424.128 graduandos de universidades federais identificou que 63,6% dos respondentes sofriam de ansiedade; 45,6% de desânimo/desmotivação; 32,7% tinham insônia/alterações no sono; 28,2% sentimento de desamparo/desespero; 23,5% declararam sentimento de solidão; 22,9% tristeza persistente; 22,1% desatenção/desorientação/confusão mental; 13,5% sentimentos de medo/pânico; 12,3% problemas alimentares; 10,8% ideação de morte e 8,5% pensamento suicida.

A relação entre a distribuição heterogênea de problemas de saúde mental e o pertencimento a diferentes classes sociais foi bem analisada em pesquisa epidemiológica que preocupou-se com a dimensão teórica de classes sociais, analisando dados sociais empíricos fragmentados como expressões da estrutura de classes^(6)^.

Coerentemente às condições de reprodução social contemporânea, este estudo adotou a compreensão de sofrimento psíquico como consequência dos característicos processos de socialização que produzem obstáculos para realização da vida. Porém, nem sempre são patológicos, a exemplo do sofrimento causado pelo luto, desde que o indivíduo encontre recursos para manter as condições de viver o cotidiano^(1)^. Portanto, atribui-se às características do modo de se produzir a vida socialmente as crescentes porcentagens de sofrimento psíquico, especialmente ansiedade e depressão^(1)^.

A natureza humana não é natural, é impregnada dos valores produzidos socialmente e um dos valores fundantes contemporâneos é a competição, que requer a desresponsabilização com o outro e o enaltecimento da moral do hedonismo. O ideário contemporâneo enaltece a autossuficiência, ilusória, fazendo os indivíduos oscilarem entre o desamparo e a onipotência^(7)^.

Este estudo parte da compreensão de que o sofrimento psíquico é resultado das experiências da vida em sociedade, que são heterogêneas, desiguais e coerentes com as características da inserção social. Considera-se as experiências entre as pessoas e entre elas e as instituições^(8,9)^, incluindo as universidades, que no Brasil passaram a abrigar uma maior heterogeneidade social de ingressantes a partir da implementação de políticas afirmativas.

Na USP, a última das universidades públicas a implementar essas políticas afirmativas, pode-se confirmar a heterogeneidade social dos graduandos pelas porcentagem de ingressantes advindos de escolas públicas; em 2024 foram 5.954 estudantes (55,4% do total de ingressantes), sendo 2.965 (27,6%) destes autodeclarados pretos, pardos ou indígenas^(10)^.

A partir dessas considerações, este estudo tomou como pressuposto que manifestações de sofrimento psíquico se distribuem heterogeneamente entre graduandos de distintos grupos sociais, e como objetivo analisar a distribuição dessas manifestações nos diferentes grupos sociais de graduandos de cursos da área da saúde da USP.

## MÉTODO

A apresentação do texto seguiu as recomendações do STROBE - Strengthening the Reporting of Observational Studies in Epidemiology^(11)^.

### Desenho do Estudo

Estudo transversal, fundamentado na epidemiologia crítica, que estuda a heterogeneidade da distribuição de doenças e agravos coerentemente com as condições de reprodução social das classes/grupos sociais^(12)^. Este estudo apresenta as manifestações de sofrimento psíquico de graduandos de cursos da área da saúde da USP, que constitui uma parcela dos resultados de pesquisa mais ampla, que incluiu graduandos de cursos das demais áreas oferecidas pela USP.

### Cenário

A coleta de dados seguiu as etapas do *Checklist for Reporting Results of Internet E-Surveys* (CHERRIES), especialmente no que diz respeito à proteção de dados^(13)^. Local: USP, com cerca de 60 mil graduandos e 12 cursos da área da saúde, no município de São Paulo (educação física e saúde, enfermagem, farmácia, fisioterapia, fonoaudiologia, gerontologia, medicina, nutrição, obstetrícia, odontologia, saúde pública, terapia ocupacional). A coleta de dados, em amostra por conveniência, foi realizada entre janeiro e agosto de 2022, após convite enviado por e-mail pelas secretarias de graduação, para todos os estudantes dos cursos, e também por divulgação de estudantes dos cursos, em grupos de whatsapp. O link do formulário foi disponibilizado em ambos os canais de comunicação. A entrada dos dados foi acompanhada via monitoramento das respostas no aplicativo de gerenciamento de pesquisas *Google Forms.*

### Participantes

O único critério para participar do estudo foi ser estudante de graduação matriculado e cursando um dos 12 cursos da área da saúde da USP, que aceitasse preencher o instrumento de coleta de dados no aplicativo *Google* Forms.

### Fonte de Dados

A coleta de dados utilizou um formulário autoaplicável, com três questionários estruturados, um deles era o de classificação social das famílias, com questões que compõem o Índice de Reprodução Social (IRS)^(14)^, resultado de pesquisa metodológica, com validação estatística, já utilizado para classificação social de famílias em outras pesquisas^(15,16)^.

O IRS parte da compreensão de que as classes sociais se reproduzem para a manutenção das relações sociais; portanto, têm condições particulares de trabalho (produção) e de vida (reprodução). O IRS capta condições consideradas relevantes na atualidade e a classificação caminha do grupo social GSI ao GSIV, indo da maior (GSI) à menor (GSIV) estabilidade nas condições de trabalho e de vida das famílias (condições de reprodução social). A exemplo de variáveis de condições de trabalho e vida que compõem o indicador, no GSI os chefes de famílias inserem-se, na maioria, em atividades relacionadas a planejamento, gerência e direção, ocupações que requerem curso preparatório para o trabalho e a quase totalidade tem casa própria; no GSII os chefes de famílias inserem-se em ocupações que não requerem curso técnico para o trabalho e parte considerável vive em casas alugadas; no GSIII os chefes de família majoritariamente estão sem inserção no mercado de trabalho, na maioria aposentados, desempregados ou afastados pelo INSS, e residem em moradias próprias; no GSIV inserem-se as famílias em condições mais instáveis, nas condições de trabalho e vida, com chefes de família inseridos em serviços gerais, que não requerem curso preparatório para o trabalho, no máximo requerem treinamento para desenvolver a atividade. Residem em moradias próprias, em condições precárias, com a quase a totalidade sem ligação à rede de esgoto oficial^(14)^.

Após pré-teste, para facilitar o autopreenchimento, acrescentou-se legenda com a Classificação Brasileira de Ocupações no formulário do IRS, para facilitar a identificação da classificação das ocupações dos chefes das famílias.

O segundo questionário foi o Self Reporting Questionnaire-20 (SRQ-20), com 20 questões sobre expressões/manifestações de sofrimento psíquico. É instrumento padronizado internacionalmente e recomendado pela OMS, pela sensibilidade e especificidade do teste^(16)^. O terceiro questionário, com 12 questões sobre expressões/manifestações de sofrimento psíquico, tem sido sistematicamente utilizado em pesquisas, obtendo resultados de centenas de milhares de graduandos de universidades federais de todo o país, pelo FONAPRACE^(5)^.

### Variáveis

Para a caracterização dos respondentes optou-se pelas variáveis: idade, gênero, local de residência, cor/etnia, processo de ingresso na universidade (vestibular, Enem/SISU, Convênio-PEC-G, transferência), curso, ano de início, bolsista ou não de programas de apoio à permanência estudantil e se é a primeira geração da família a cursar o ensino superior.

Para a caracterização do IRS: qualificação do trabalho do chefe da família, realização de curso preparatório para o trabalho, (in)formalidade no trabalho ou desemprego; propriedade da habitação, acesso legalizado à água, esgoto e energia elétrica, número de cômodos para dormir, pagamento de IPTU e ida ao culto como forma de lazer.

Para identificar manifestações de sofrimento psíquico, foram adotadas as mesmas variáveis utilizadas pelo FONAPRACE (2019): ansiedade; tristeza persistente; timidez excessiva; medo/pânico; insônia ou alterações significativas de sono; sensação de desamparo/desespero/desesperança; sensação de desatenção/ desorientação/ confusão mental; problemas alimentares; desânimo/falta de vontade de fazer as coisas; sentimento de solidão; ideia de morte, e pensamento suicida^(5)^ e adotadas as variáveis do *Self Reporting Questionnaire-20* (SRQ-20), sobre a presença de sintomas físicos e psíquicos, contendo escala dicotômica (sim/não) para cada uma das suas questões^(17)^.

### Tamanho da Amostra

A determinação da amostra foi feita por meio da relação entre manifestações de sofrimento psíquico e grupo social, considerando a população da pesquisa que deu origem a este estudo. O valor de tamanho de efeito foi considerado com base nas informações da amostra piloto, com 33 graduandos. Nessa amostra observou-se um valor de W de 0,65; visando amostra mais conservativa, adotou-se o valor de W = 0,40. Desta forma, foi calculada uma amostra de 108 graduandos para cada uma das 4 áreas (Saúde, Ciências Biológicas, Ciências Humanas e Ciências Exatas), totalizando uma amostra efetiva de 432 alunos, para se detectar um tamanho de efeito (effect size) de 0,4, num teste de Qui-Quadrado com 3 graus de liberdade a um nível de significância de 5% e poder de 95%. A amostra não é probabilística. A partir desse cálculo é que definiu-se para este estudo a amostra de 108 graduandos da área da saúde.

### Variáveis Quantitativas

Foram ajustados modelos de regressões logísticas, tendo como variável dependente manifestação de sofrimento psíquico e como variáveis explicativas, gênero, idade, cor, cidade, residência na USP, ano de início do curso e grupo social. No modelo de regressão logística os coeficientes exponenciados são interpretados como razão de chances. Neste estudo, a chance consiste no quociente entre a probabilidade de um aluno apresentar manifestação de sofrimento psíquico e a probabilidade de não apresentar tal condição.

### Métodos Estatísticos

A análise foi realizada utilizando-se o pacote estatístico SPSS 20.0 4^1^ e STATA 17^2^. A consistência interna global entre os itens do SRQ20 foi analisada via coeficiente Alpha de Cronbach; quanto mais próximo de 1 é maior a consistência entre os itens da escala ou domínio. Para todos os testes estatísticos foi utilizado um nível de significância de 5%.

O resultado esperado é a distribuição heterogênea de sofrimentos psíquicos entre estudantes dos diversos grupos sociais, com mais chance de manifestações entre estudantes de grupos sociais com maior instabilidade nas condições de trabalho e de vida das famílias.

### Aspectos Éticos

O estudo foi aprovado por Comitê de Ética em Pesquisa (parecer nº 246/2021/CPq/EEUSP). A participação após anuência formal do Termo de Consentimento Livre e Esclarecido foi on-line, via preenchimento de formulário eletrônico. No final desse instrumento constava link com o mapa de recursos de saúde mental da USP, caso o respondente sentisse necessidade de ajuda (https://mapadesaudemental.prip.usp.br/).

## RESULTADOS E ANÁLISE

Foram 188 os respondentes, de 11 cursos, sem participantes do curso de educação física e saúde. Destes, 144 (77,4%) declararam-se do gênero feminino e 121 (64,7%) de cor branca.

A média de idade dos respondentes foi 21,5 anos (DP = 4,9 anos), com idade mínima de 17 anos e máxima de 65 anos, sendo que 94 (50%) tinham idades entre 19 e 22 anos.

Por referência ao local de residência, 131 (69,7%) residiam no município de São Paulo e apenas 9 (4,8%) dos respondentes residiam no conjunto residencial da USP (CRUSP).

Quanto à classificação social, 95 (50,5%) das famílias foram classificadas no grupo social I (GSI), em que os chefes de família têm maior estabilidade nas condições de trabalho e de vida, por referência aos demais três grupos sociais (GS). No grupo social II (GSII) foram 49 (26,1%) dos respondentes; as famílias classificadas como grupo social III (GSIII) eram 26 (13,8%) e 18 (9,6%) eram as do grupo social IV (GSIV). Devido à menor percentagem das famílias classificadas como GSIII e GSIV estas foram agrupadas, correspondendo então a 23,4% (44) dos respondentes. A mesma estratégia foi usada por estudo que analisou o desenvolvimento infantil em diferentes grupos sociais^(18)^.

A análise identificou 133 (70,7%) respondentes com manifestações de sofrimento psíquico e constatou a associação entre GS e manifestação de sofrimento psíquico (p = 0,031). Entre os respondentes do GSII, 149 (79,6%) apresentavam sofrimento psíquico, o mesmo número, 149 (79,5%), entre os respondentes de famílias GSIII/GSIV e 116 (62,1%) entre respondentes do GSI, como pode ser observado na Tabela 1.

**Tabela 1:** Distribuição do sofrimento psíquico por características e grupos sociais dos estudantes dos cursos da saúde da USP – São Paulo, SP, Brasil, 2022.

	sofrimento psíquico (SRQ-20 ≥ 7)		p
	Não	Sim	
**Gênero, N (%)**			0,496^a^
Feminino	39/144 (27,1)	105/144 (72,9)	
Masculino	14/39 (35,9)	25/39 (64,1)	
Não binário	1/3 (33,3)	2/3 (66,7)	
**Idade (anos), Média ± DP**	21,9 ± 7,6	21,4 ± 3,2	0,610^c^
**Cor/Etnia, N (%)**			0,436
Branca	40/121 (33,1)	81/121 (66,9)	
Parda	7/35 (20,0)	28/35 (80,0)	
Preta	4/18 (22,2)	14/18 (77,8)	
Amarela/indígena	4/13 (30,8)	9/13 (69,2)	
**Cidade, N (%)**			0,107
Município de São Paulo	40/131 (30,5)	91/131 (69,5)	
Outro Município do Estado de São Paulo	9/45 (20,0)	36/45 (80,0)	
Município de outros estados da federação	6/12 (50,0)	6/12 (50,0)	
**Reside na Univ, N (%)**			0,288^a^
Não	54/179 (30,2)	125/179 (69,8)	
Sim	1/9 (11,1)	8/9 (88,9)	
**Ano de início do curso**			0,099
2018	7/27 (25,9)	20/27 (74,1)	
2019	6/28 (21,4)	22/28 (78,6)	
2020	8/31 (25,8)	23/31 (74,2)	
2021	7/37 (18,9)	30/37 (81,1)	
2022	27/65 (41,5)	38/65 (58,5)	
**Grupo Social, N (%)**			0,031
GSI	36/95 (37,9)	59/95 (62,1)	
GSII	10/49 (20,4)	39/49 (79,6)	
GSIII/GSIV	9/44 (20,5)	35/44 (79,5)	

p -nível descritivo do teste de Qui-Quadrado

Exato de Fisher^(a)^

e t de Student^(c)^.

Essa associação não foi estatisticamente significativa para as categorias cor/etnia, idade e gênero, cidade de origem, residir na moradia universitária e sofrimento psíquico, como observa-se na Tabela 1. As variáveis gênero, cor/etnia não foram respondidas pela totalidade dos respondentes.

Após ajustes de regressão logística, verifica-se que permaneceu significante no modelo final o pertencimento ao GS (p = 0,034). Dessa forma, observou-se que as chances de sofrimento psíquico (SRQ20 ≥7) em graduandos dos grupos GSII e GSIII/GSIV foram similares; porém foram maiores do que entre as do grupo GSI (cerca de 2,4 vezes maior), expresso na Tabela 2.

**Tabela 2:** Chance dos estudantes dos cursos da área da saúde da USP de apresentarem sofrimento psíquico segundo gênero, idade, cor, cidade, residir na moradia estudantil, ano de início do curso e grupo social – São Paulo, SP, Brasil, 2022.

	Modelo univariado		Modelo multivariado inicial		Modelo final	
	RC (IC95%)	p	RC (IC95%)	p	RC (IC95%)	p
**Gênero (ref. Feminino)**		0,555		0,931		−
Masculino	0,66 (0,31–1,40)	0,284	0,91 (0,38–2,15)	0,822	−	−
Não binário	0,74 (0,07–8,42)	0,810	0,66 (0,05–8,88)	0,751	−	−
**Idade (anos)**	0,98 (0,92–1,04)	0,494	0,95 (0,88–1,02)	0,157	−	−
**Cor/Etnia (Ref. = Branca)**		0,444		0,610	−	−
Parda	1,98 (0,79–4,91)	0,143	1,95 (0,71–5,34)	0,192	−	−
Preta	1,73 (0,53–5,59)	0,361	1,25 (0,32–4,82)	0,749	−	−
Amarela/Indígena	1,11 (0,32–3,83)	0,867	0,88 (0,23–3,33)	0,847	−	−
**Cidade (ref. = Município de São Paulo)**		0,119		0,177	−	−
Outro Município do Estado de São Paulo	1,76 (0,77–3,99)	0,177	2,05 (0,85–4,97)	0,111	−	−
Município de outros estados da federação/Outro país	0,44 (0,13–1,45)	0,176	0,60 (0,14–2,58)	0,488	−	−
**Reside na Universidade**	3,46 (0,42–28,31)	0,248	3,05 (0,31–29,81)	0,337	−	−
**Ano de início do curso (ref. = 2019)**		0,109		0,077		−
2015 a 2018	0,78 (0,22–2,71)	0,695	0,72 (0,17–3,01)	0,653	−	−
2020	0,78 (0,23–2,63)	0,693	0,64 (0,15–2,64)	0,535	−	−
2021	1,17 (0,35–3,96)	0,802	0,89 (0,21–3,74)	0,871	−	−
2022	0,38 (0,14–1,07)	0,068	0,25 (0,07–0,92)	0,036		
**Grupo Social (ref. = GSI)**		0,034		0,132		0,034
GSII	2,38 (1,06–5,34)	0,036	2,17 (0,87–5,43)	0,097	2,38 (1,06–5,34)	0,036
GSIII/GSIV	2,37 (1,02–5,51)	0,044	2,16 (0,81–5,73)	0,122	2,37 (1,02–5,51)	0,044

RC: razão de chances (Odds ratio). IC: Intervalo de confiança. N = 184 e N = 188, respectivamente para os modelos multivariados inicial e final, Teste de adequacidade do ajuste de Hosmer e Lemeshow: modelo multivariado inicial (p = 0,860) e final (p = 1,000).

Identificou-se significância estatística para a associação entre GS e sentimento de timidez excessiva (p = 0,046) e entre GS e sensação de desamparo/desespero/desesperança (p = 0,026). No GSII e GSIII/GSIV as porcentagens maiores foram de sentimento de timidez excessiva, 37,6% (35 respondentes), enquanto que no do GSI a timidez excessiva foi referida por 24,2% (23 respondentes). A sensação de desamparo/desespero/desesperança foi citada por 64,5% (60 respondentes) dos grupos GSII, GSIII/GSIV e por 48,4% (46) do GSI.

## DISCUSSÃO

Respondentes deste estudo foram predominantemente do gênero feminino, brancos e de famílias do GSI. A predominância do gênero feminino em cursos da área da saúde também foi identificada em outras pesquisas(19-21).

O perfil de maioria branca encontrado neste estudo acompanha a tendência nacional nas universidades, que têm cerca de 20% mais estudantes brancos do que a sociedade brasileira como um todo^(22)^. Resultados de estudo realizado em três universidades federais mineiras identificaram que, embora a maioria fosse branca nos cursos da área da saúde, nos cursos de Biomedicina, Educação Física e Enfermagem a maioria era não-branca^(22)^.

Na amostra deste estudo as famílias classificadas como GSI e GSII juntas representaram 76,6% dos respondentes, o que confirma que embora tenham sido adotadas políticas afirmativas no Brasil, a expansão de vagas no ensino superior ainda não garante a igualdade social no acesso^(23)^. O ingresso na universidade ainda é marcado por profundas desigualdades, jovens de classes sociais mais estáveis têm chances muito maiores^(24)^.

Além disso, as dificuldades não terminam com o ingresso na universidade, foi identificada associação entre origem social e sentimento de pertencimento, qualidade da experiência na universidade e características da adaptação universitária^(25)^. A universidade é também um espaço social de vivência de sofrimento psíquico^(26)^ pois, como instituição social, reproduz valores sociais hegemônicos. Desta forma, o ingresso em universidades públicas ao mesmo tempo que é um potencial de fortalecimento para as famílias, é potencial de desgaste, de sofrimento para os graduandos.

A porcentagem de graduandos com sentimento de timidez excessiva e sensação de desamparo/desespero/desesperança foi maior entre graduandos dos grupos de menor estabilidade (GSII, GSIII/GSIV), quando comparada a de respondentes do GSI, grupo social de maior estabilidade.

Nesse contexto, a universidade precisa preparar-se para receber graduandos com condições sociais heterogêneas. O que tem-se presenciado, ao contrário, são graduandos ingressando em instituição que valoriza a cultura meritocrática, que produz neles o sentimento de incapacidade^(27)^.

Embora seja inegável que o ingresso na universidade é um potencial de fortalecimento para os graduandos e suas famílias, também representa potencial de desgaste, mais intenso para os de grupos sociais com maior instabilidade. Some-se a isso a identificação neste estudo de que a possibilidade de contar com redes de apoio decresce à medida que se caminha do GSI ao GSIV, sendo os estudantes do GSI os que contam com a maior rede de apoio, também identificada em outros estudos^(14,28)^ como fortalecimentos à saúde mental.

Na atualidade o cotidiano universitário reproduz valores sociais hegemônicos, como a competitividade e o sucesso individual, que produzem sentimento de desamparo^(8)^, opostamente às vivências coletivas e solidárias, que reforçam o sentimento de pertencimento. No entanto, a universidade tem condições de propor ações para o enfrentamento dessa lógica de enaltecimento de conquistas individuais^(18)^, para enfrentar o sofrimento psíquico de estudantes para além de sua dimensão individual, a partir dos processos sociais na sua gênese^(9)^.

Para o enfrentamento do sofrimento psíquico de graduandos pode-se recorrer a políticas universitárias: para estabelecer e dar suporte ao fortalecimento da saúde mental; identificar necessidades em saúde mental dos estudantes e dar respostas a elas, no âmbito dos cursos; integrar e ampliar a oferta de programas de atenção à saúde mental e proporcionar acesso dos estudantes a eles; promover programas educativos e estratégias comunicacionais sobre o sofrimento psíquico na contemporaneidade e formas de enfrentamento, para que os estudantes possam encontrar práticas fortalecedoras; monitoramento constante e avaliação contínua das necessidades em saúde mental de estudantes^(29)^. Para lograr a plena integração dos universitários à vida acadêmica, a formação dos graduandos deve contar com estruturas sólidas de suporte, envolvendo todos os setores institucionais. A importância da sociabilidade estudantil deve fazer parte da formação dos trabalhadores das universidades^(30)^.

## CONCLUSÃO

Os resultados deste estudo confirmaram o pressuposto de que existe distribuição heterogênea de sofrimento psíquico, atestado pelo SQR-20. Os resultados mostraram que os estudantes dos grupos GSII e GSIII/GSIV manifestaram cerca de 2,4 vezes mais chances de apresentarem sintomas de sofrimento psíquico em relação ao grupo GSI. O sentimento de timidez excessiva e sensação de desamparo/desespero/desesperança foram mais recorrentes nos grupos GSII e GSIII/GSIV.

Embora a USP venha instaurando ações em resposta a necessidades relacionadas a sofrimento psíquico, a exemplo do projeto ECOS (Escuta, Cuidado e Orientação em Saúde Mental), entre outras ações da Pró Reitoria de Inclusão e Pertencimento (PRIP), para aprofundar as iniciativas já existentes defende-se a priorização de recursos materiais e imateriais da universidade para aprimorar a infraestrutura de moradias e das instituições de ensino; para aprimorar a articulação entre as ações implementadas para enfrentamento do sofrimento psíquico, a começar por processos de identificação dos GS dos estudantes e das manifestações do sofrimento, para fortalecer projetos voltados à prevenção e ao cuidado, tomando como premissa as raízes da produção do sofrimento.

A limitação do estudo encontra-se nos possíveis vieses, uma vez que a amostra foi constituída por conveniência; portanto, pode ter havido tendência de respostas de graduandos mobilizados pela temática, por estarem vivenciando sofrimento psíquico.

Este estudo limitou-se à investigação com estudantes da área de saúde. Para ampliar a análise, são necessários estudos entre graduandos de outras áreas.

## Footnotes

1 IBM Corp. Released 2011. IBM SPSS Statistics for Windows, Version 20.0. Armonk, NY: IBM Corp.

2 StataCorp. 2021. Stata Statistical Software: Release 17. College Station, TX: StataCorp LLC.
